# Repair of Long Nerve Defects with a New Decellularized Nerve Graft in Rats and in Sheep

**DOI:** 10.3390/cells11244074

**Published:** 2022-12-16

**Authors:** Estefanía Contreras, Sara Traserra, Sara Bolívar, Joaquim Forés, Eduard Jose-Cunilleras, Felix García, Ignacio Delgado-Martínez, Sandra Holmgren, Raimund Strehl, Esther Udina, Xavier Navarro

**Affiliations:** 1 Department of Cell Biology, Physiology and Immunology, Institute of Neuroscience, Universitat Autònoma de Barcelona, 08193 Bellaterra, Spain; 2 Integral Service for Laboratory Animals (SIAL), Faculty of Veterinary, Universitat Autònoma de Barcelona, 08193 Bellaterra, Spain; 3 Centro de Investigación Biomédica en Red sobre Enfermedades Neurodegenerativas (CIBERNED), 28031 Madrid, Spain; 4 Hand and Peripheral Nerve Unit, Hospital Clínic i Provincial, Universitat de Barcelona, 08036 Barcelona, Spain; 5 Department of Animal Medicine and Surgery, Universitat Autònoma de Barcelona, 08193 Bellaterra, Spain; 6 VERIGRAFT AB, Arvid Wallgrens Backe 20, 41346 Göteborg, Sweden

**Keywords:** nerve regeneration, autograft, allograft, decellularization, sheep

## Abstract

Decellularized nerve allografts (DC) are an alternative to autografts (AG) for repairing severe peripheral nerve injuries. We have assessed a new DC provided by VERIGRAFT. The decellularization procedure completely removed cellularity while preserving the extracellular matrix. We first assessed the DC in a 15 mm gap in the sciatic nerve of rats, showing slightly delayed but effective regeneration. Then, we assayed the DC in a 70 mm gap in the peroneal nerve of sheep compared with AG. Evaluation of nerve regeneration and functional recovery was performed by clinical, electrophysiology and ultrasound tests. No significant differences were found in functional recovery between groups of sheep. Histology showed a preserved fascicular structure in the AG while in the DC grafts regenerated axons were grouped in small units. In conclusion, the DC was permissive for axonal regeneration and allowed to repair a 70 mm long gap in the sheep nerve.

## 1. Introduction

Peripheral nerve injuries results in partial or total loss of the sensory, autonomic and motor functions dependent on the injured nerve, with important consequences for the quality of life of affected patients [1]. It is estimated that more than 300,000 persons are affected by traumatic nerve injury every year in North America and Europe [2,3]. Although peripheral neurons are able to regenerate after axotomy, and eventually reinnervate target organs, functional recovery is often unsatisfactory, especially following severe injuries [4,5]. Poor functional outcomes generally stem from long regenerative distances coupled with a relatively slow rate of axonal regeneration (~1–2 mm/day), creating prolonged periods of denervation that ultimately limit the regenerative capacity of the distal nerve structure [6,7]. Therefore, a special challenge regarding recovery after peripheral nerve injury are long distance nerve defects and proximal injuries in long limbs.

When the length of the gap created by a nerve injury is too long to allow apposition and direct suture without tension, nerve grafts are usually employed for repairing. The graft between the stumps of a transected nerve offers mechanical guidance, and the Schwann cells of the graft and their basal lamina play an essential role in promoting axonal growth [8]. The autologous nerve graft represents the ‘‘gold standard’’ surgical treatment for complex peripheral nerve injuries in the clinic; however, autografts present a number of limitations. Apart from the paucity of expendable nerve tissue, harvesting autologous nerves results in significant donor site morbidity, increased risk of infection, and longer intraoperative times [9]. The alternative use of allografts has been discouraging, since the immune rejection directed against Schwann cells and myelin sheaths of the graft impedes axonal regeneration [10]. Processed acellular nerve allograft has been promoted as a potential replacement [9,11]. If adequately decellularized, such grafts do not induce immune response, whereas the internal nerve structure, including endoneurial tubules, basal lamina and extracellular matrix (ECM) components, remains supporting axonal regeneration, despite the absence of the activated Schwann cells [8].

Allograft and autograft repair of nerve defect injury in small animals have indicated that regeneration is possible on critical length nerve gaps [12,13]. Even though the anatomy of rodent nerves has been well studied and is similar to that of humans [14], the important difference in size between these species compared to humans limits the translation of the results obtained, since axonal regeneration is faster in smaller animals and relatively short gaps can be produced in them [15,16]. Indeed, it is not clear if the mechanisms involved in peripheral nerve trauma, repair and regeneration are closely conserved across species [6,16,17]. In order to achieve results that may be applicable to humans, a translational large animal model for peripheral nerve surgery is important [6,17,18]. A valid animal model for nerve regeneration studies should have similar anatomy and length of the nerves to humans and follow a similar regeneration rate after nerve injury. Sheep have emerged as one of the most adequate large animals for pre-clinical studies on a variety of peripheral nerves [18,19,20,21,22,23]. Thus, experimental studies in small and large animal models are fundamental to optimize the decellularization process and to evaluate the regenerative capacity of novel allografts. In this study, we assessed a newly prepared decellularized nerve graft used for repairing a critical gap of 15 mm of the sciatic nerve in rats, and given the obtained success, we extended to a comparative assessment of nerve regeneration along a nerve gap of 70 mm length in the peroneal nerve of adult sheep repaired with the new decellularized nerve graft, provided by VERIGRAFT AB, or with an autograft as the gold standard method of repair.

## 2. Materials and Methods

### 2.1. Nerve Decellularization

Rat sciatic nerves were harvested from five euthanized Sprague Dawley rats under aseptic conditions. Specimens of sheep nerves for the decellularized allografts were obtained from four donor sheep from the colony of Servei de Granges i Camps Experimentals (SGiCE) of the Universitat Autònoma de Barcelona (UAB). Peroneal nerves were harvested from euthanized sheep under aseptic conditions. The harvested nerves were stored in phosphate buffered saline (PBS) plus antibiotic-antimycotic agents, maintained at 4 °C and shipped to VERIGRAFT AB. 

For decellularization, the nerves were thawed and agitated in NaCl (Fisher Scientific, Göteborg, Sweden) for 24 h, PBS (Medicago, Uppsala, Sweden) for 6 h and Deoxyribonuclease (DNase; VWR, Stockholm, Sweden) overnight. The nerves were washed 3 × 5 min in H_2_O after NaCl and DNase. This process was repeated three times. Then the nerves were washed in PBS for 24 h. After decellularization, samples were taken for DNA quantification and histology before peracetic acid sterilization and final washes in PBS under sterile conditions. The sterilized decellularized nerves were shipped in PBS at 2–4 °C. One nerve segment was decellularized for each animal to be transplanted. DNA was extracted from 10–25 mg wet tissue with the DNeasy Blood & Tissue kit (Qiagen, Mettmann, Germany) and quantified with the Qubit dsDNA HS assay kit (Invitrogen, Waltham, MA, USA), according to the manufacturer’s instructions. DNA concentration was calculated using the DNA standard supplied with the Qubit dsDNA HS assay kit. 

After decellularization, a small segment of each decellularized nerve was fixed in paraformaldehyde 4% for 4 h at room temperature (RT) and then transferred to PBS with sucrose 30% for 24 h. Samples were cryo-embedded with OCT and serially cut in 15 µm thick transverse sections with a cryotome. The sections were blocked and permeabilized with 0.3% Triton-X100 and 10% normal donkey serum and/or goat donkey serum for 1 h. Then, slides were incubated overnight at 4 °C with primary antibodies against anti-rabbit S-100 protein (S100; to label Schwann cells; 1:50; 22520-DiaSorin), anti-chicken neurofilament (NF200; myelinated axons; 1:400; AB5539-Millipore), anti-rabbit non-collagenous connective tissue glycoprotein (laminin; 1:500; AHP420-Biorad), and with myelin stain (Fluoromyelin; 1:300; F34651-Invitrogen), all diluted in 0.1% Triton-PBS. Following three washes in PBS, the sections were incubated with secondary antibodies conjugated to Alexa Fluor 488 goat anti-chicken (1:200; A11039-Invitrogen) and Alexa Fluor 594 goat anti-rabbit (1:200; A21207-Invitrogen) for 1 h. After two washes with PBS, samples were incubated with DAPI stain for nuclei (1:100; D9564-10MG-Sigma) diluted in PBS for 2 min. After three more washes in PBS, immunolabeled sections were cover-slipped with Fluoromount (Sigma-Aldrich, St. Louis, MI, USA) and viewed under epifluorescence microscopy (Olympus BX51).

All the procedures were approved by the Ethics Committees of the UAB and Generalitat de Catalunya, and followed the European Community Council Directive 2010/63/EU of the European Parliament on the protection of animals used for scientific purposes.

### 2.2. Rat Study

For the implantation, ten female Sprague Dawley rats (12 weeks of age) were randomly assigned into two different experimental groups: autograft (AG) (*n* = 5) or decellularized rat allograft (DRA) (*n* = 5). Animals were anesthetized with ketamine (75 mg/kg) and medetomidine (0.01 mg/kg) intraperitoneally. A skin incision was made on the right hindlimb and the biceps femoris muscle was incised to expose the sciatic nerve. A 15 mm in length was removed and then, in the AG group, the same nerve was sutured again maintaining the same orientation. In the DRA group, the nerve removed was substituted by a 15 mm length decellularized rat allograft. In both cases, the proximal and distal nerve stumps were sutured by 10-0 nylon epineural sutures. Postoperative care included amitriptyline (20 mL/L) in drinking water to prevent autotomy [24] and buprenorphine (0.03 mg/kg subcutaneously) to treat postoperative pain. 

Reinnervation of target muscles were assessed by monthly noninvasive nerve conduction tests until the end time, set at 120 days post injury (dpi). Under general anesthesia, the rat was placed on a warm plate, and the sciatic nerve was stimulated with transcutaneous needle electrodes placed at the sciatic notch using a Sapphire 4M electromyograph (EMG) (Medelec, Vickers, Surrey, U.K.), by delivering single pulses of 0.1 ms of increasing intensity. The compound muscle action potential (CMAP) was recorded from the tibialis anterior (TA), gastrocnemius (GM) and plantar interosseus (PL) muscles with small needle electrodes transcutaneously placed on the active muscle belly and the reference in the fourth toe [25,26]. The ground electrode was placed at the knee. The contralateral hindlimb was used as a control since there are not significant changes in electrophysiological properties of the contralateral limb after a unilateral nerve lesion [25].

At the end of the study, animals were euthanized by an overdose of pentobarbital (200 mg/kg i.p.) and samples from the sciatic nerve were harvested. Samples were fixed in paraformaldehyde 4% and then, divided in three different parts. The proximal and distal segments, including suture levels were post-fixed in 3% glutaraldehyde-3% paraformaldehyde in cacodylate-buffer solution (0.1 M, pH 7.4) at 4 °C for 48 h. To evaluate the microstructure of the nerve, samples were embedded in epon resin. Semithin 0.5 μm sections were stained with 1% toluidine blue and visualized with a light microscope (Olympus BX40). The cross-sectional area of the whole nerve was measured at 40X. Images were obtained at 1000X from fields chosen by systematic sampling of squares, representing at least 30% of the transverse area of the nerve [27,28]. Counts of the number of myelinated nerve fibers were conducted using ImageJ software (version 1.52d). Only myelinated fibers whose contour was completely within each field photograph or cut at two borders of the square were counted. The total number of myelinated fibers per nerve was obtained from the fiber density of the sampled fields and the total transverse endoneurial area. The middle segment of the sciatic nerve was processed for immunolabeling as explained above for axons (NF200), Schwann cells (S100), laminin and macrophages (anti-goat iba 1; ionized calcium-binding adapter molecule 1; 1:500; 19-19741-Rafer). Samples were washed and incubated with secondary antibodies Alexa Fluor 488 goat anti-chicken (1:200; A11039-Invitrogen), Alexa Fluor 488 donkey anti-goat (1:200; A11055-Invitrogen) and Alexa Fluor 594 goat anti-rabbit (1:200; A21207-Invitrogen) diluted in PBS-Triton 0.3%. Finally, sections were cover-slipped with Fluoromount containing DAPI (1:10,000; Sigma-Aldrich). Sections were visualized with an epifluorescence microscope (Olympus BX51) for qualitative assessment of the regeneration process. Observations were focused on the relationship between axons and Schwann cells and the number of infiltrating macrophages.

### 2.3. Sheep Study

Ten female ripollesa sheep (Ovis aries), weighing 55–75 Kg, from the animal facility of SGiCE of the UAB were used. The sheep were divided in two experimental groups of 5 animals each, according to the repair procedure performed: autograft and decellularized allograft. A blood sample was taken before the surgery and at the end of the study to perform hematological and biochemical standard analyses, to ensure that the sheep were in good health state. A general clinical assessment was performed once a week until the end of the study.

### 2.4. Surgical Procedure

The animals were fasted 16 h prior to the surgery to reduce the ruminal content and prevent deviant swallowing. Animals were sedated by midazolam (0.2 mg/Kg) and morphine (0.4 mg/Kg i.m.). Anesthesia was induced by propofol (4 mg/Kg i.v.) and maintained by isofluorane 2% in 2 L/min oxygen administered with a pressure-controlled ventilator. Analgesia was provided by diazepam (0.5 mg/Kg i.v.), and an antibiotic dose of cefazoline (20 mg/Kg i.v.). Fluid therapy was applied with Ringer solution infusion (10 mL/Kg/h).

**Figure 1:**
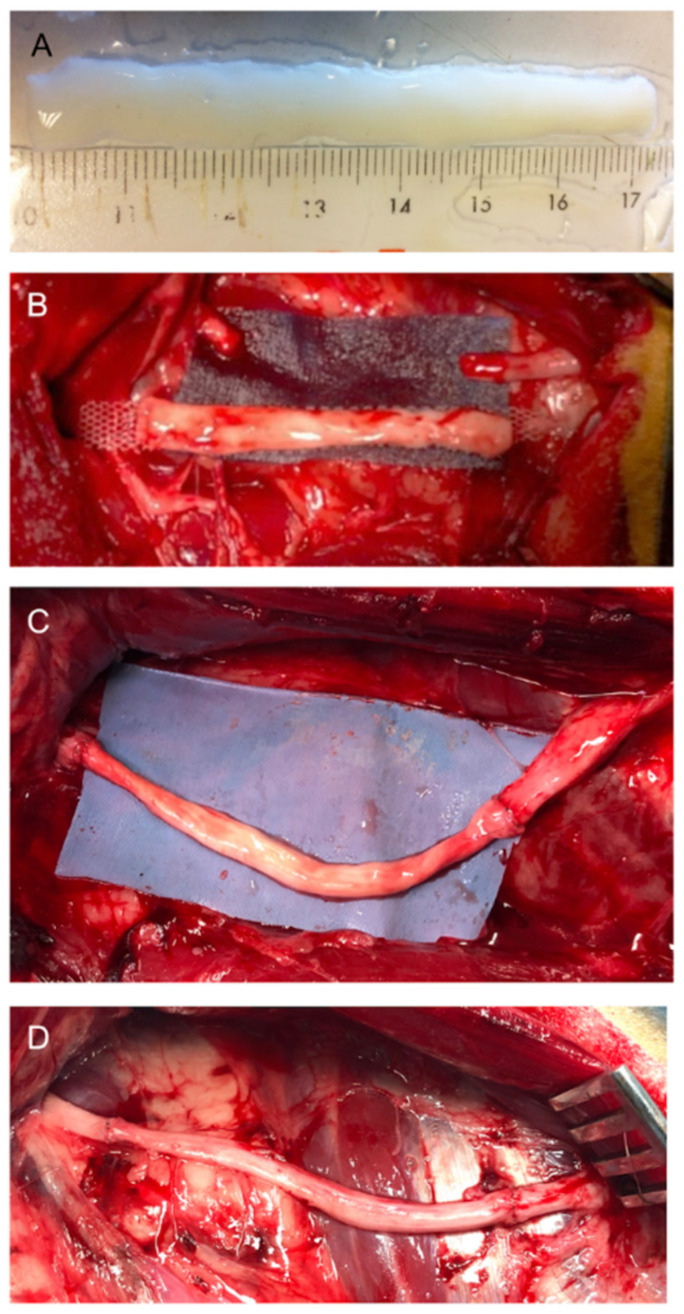
Decellularized nerve allograft (**A**) was trimmed into 7-cm nerve long and then it was placed into the gap created in the common peroneal nerve (**B**). The decellularized nerve allograft (**C**) or the same nerve segment resected used as autograft (**D**) were sutured bridging the gap.

The operative procedure was conducted using a sterile technique with the sheep in a left lateral decubitus position on an operating table. On the right hindlimb, the peroneal nerve was exposed using a longitudinal lateral skin incision along the thigh followed by splitting of the semitendinous and biceps femoris muscles. Under the operating microscope, the common peroneal nerve was resected 1 cm above the iliac vein to create a 7 cm gap. The repair was conducted by bridging the two nerve stumps with a decellularized nerve allograft or with an autograft (Figure 1). The resected nerve segment was used as autograft maintaining the original orientation. The decellularized nerve graft was easy to handle and suture, without notable difference compared with the nerve autograft. The nerve stumps were sutured to each end of the graft by means of 8/0 epineural sutures. The suture resistance was tested against light stretching. The incision was closed in layers and disinfected with chlorhexidine and Aluspray^®^. After the surgery, the animals were allowed to recover in couples in the animal’s facility. During the post-operative period, analgesia was provided twice a day with buprenorphine (0.01 mg/Kg s.c.) for 2 days and once a day with meloxicam (0.2 mg/Kg s.c.) for 3 days.

### 2.5. Functional Tests

At monthly intervals, animals were tested for evidence of hindlimb functional recovery. Each parameter was assessed in a semiquantitative arbitrary scale from 0 (no deficit), −1 (partial deficit or functional loss) to −2 (complete loss of response in the maneuver tested). Locomotion was assessed qualitatively while freely walking in the stable, paying particular attention to the foot-drop position of the operated hindlimb (0 = normal walking; −1 = a few failures; −2 = foot drop in mots steps). The TA muscle mass was assessed by manual palpation comparing both the denervated and control side (0 = similar mass; −1 = slight reduction; −2 = marked reduction). The proprioceptive response was tested by the capability of replacing the hoof from a plantarflexion position forced by the experimenter to a plantar support position (0 = consistent response; −1 = one failure; −2 = two or 3 failures). The withdrawal reflex of the limb was evaluated by pinching with a hemostat the skin of the dorsum of the paw at three sites between the ankle and the hoof (Figure S1), proximal, middle and distal. Each site was tested twice in the same session and separately scored as: 0 = fast and strong withdrawal response of the limb; −1 = weak response or not consistent in the 2 trials; −2 = no response in 2 trials.

### 2.6. Electrophysiological Tests

Motor nerve conduction tests were performed at 5, 6.5 and 9 months after the operation under general anesthesia (diazepam 0.25 mg/Kg and ketamine 5 mg/Kg i.v.). An EMG apparatus (Sapphire 4ME, Vickers, Surrey, U.K.) was used for these tests. The sciatic nerve was stimulated, at supramaximal intensity, at the sciatic notch with a pair of percutaneous needle electrodes, and the compound muscle action potential (CMAP) was recorded from the TA muscle with monopolar needle electrodes, the active electrode on the muscle belly, and the reference at the distal tendon. Moreover, free-running EMG recordings were made to detect fibrillation potentials, as a sign of muscle denervation. The contralateral hindlimb was tested at the same session and its values used as control.

### 2.7. Ecographic Evaluation of Hindlimb Muscles

Ultrasound examinations were performed at 6.5 and 9 months after the electrophysiological tests, while animals were anesthetized, using a MyLab^®^ Gamma (Esaote, Genova, Italy) device and a linear ultrasound probe. The cross-sectional area and perimeter of the TA muscle was determined using a B-mode ultrasound, employing a 15 MHz linear transducer, and optimizing the image at a depth of 3 cm and focal point at 1.5 cm. To optimize image acquisition and good skin contact, the hair over the area of interest was clipped and the skin cleaned with water and mild soap, and acoustic gel was used. In preliminary studies, we established that the point of interest to standardize image acquisition was the cranial aspect of the crus at a midpoint between the tibial crest and the tuber calcanei (Figure S2).

### 2.8. Histological Evaluation

After performing the functional test 9 months after injury and when still anesthetized, animals were euthanized using an intravenous injection of Euthasol (400 mg/Kg i.v.). Nerve segments extending at least 1 cm from the proximal suture and 2 cm from the distal suture of the graft were harvested and fixed in paraformaldehyde 4% for 7 days at 4 °C. TA muscles were taken and weighed, and samples of TA muscles and of the skin of the dorsum of the paw were obtained and fixed in paraformaldehyde 4% for 7 days at room temperature.

After fixation, the peroneal nerves were divided into 5 different sections (see Section 3.3.4), and each section was further divided in two halves. The first half of Section 2 from the middle of the graft and Section 4 from the distal end were embedded in paraffin and 5 µm thick cross sections were obtained with a microtome. Samples from the midpoint of the graft were deparaffinized and stained with hematoxylin at 10% in absolute ethanol (Hematoxylin cryst. 1.04302.0100, Sigma Aldrich) and eosin at 0.5% in distilled water (Eosin Y, 1.15935.0100, Sigma Aldrich) to visualize the general structure of the nerve. Briefly, the samples were hydrated in H_2_O for 10 min, then, stained for 5 min in hematoxylin. After two washes in H_2_O, samples were introduced in 70% ethanol with 1% of hydrochloric acid for 30 s. Samples were washed again and stained with eosin for 5 min. Finally, samples were dehydrated, and mounted in gelatinized glass slides. Other sections were dewaxed and processed for immunohistochemistry against S-100 protein to label Schwann cells, and neurofilament NF200 to label myelinated axons, as explained above. Following washes, the sections were incubated with secondary antibodies bound to Alexa Fluor 488 and Alexa Fluor 594, mounted on gelatinized slides and viewed under epifluorescence microscopy. Quantitative analysis was performed with sets of images obtained at 1000X by measuring the cross-section area of the nerves and grafts, and then counting the number of NF200 labeled axons in systematically selected fields, with at least 8 representative fields comprising more than 30% of the transverse area, under epifluorescence microscopy (Olympus BX51) using ImageJ software.

The second half of Section 2 from the middle of the graft and Section 4 from the distal nerve were postfixed in 3% paraformaldehyde and 3% glutaraldehyde in phosphate-buffered saline. The nerve segments were postfixed in osmium tetroxide, dehydrated in a series of ethanol, and embedded in epon resin. Semithin 0.5 µm thick sections were stained with toluidine blue, and representative images were taken by light microscopy.

TA muscle and skin samples were also processed for paraffin embedding. Samples were stained with hematoxylin and eosin, as explained above for the nerve samples, to visualize the general structure and to evaluate qualitatively the areas showing denervation atrophy.

### 2.9. Data Analysis

Data are expressed as mean ± standard error of the mean (SEM). The results of functional tests and histology were analyzed by the Student’s *t* test and a two-way ANOVA after checking for normal distribution, using GraphPad Prism 8 software. A *p* < 0.05 was considered as significant.

## 3. Results

### 3.1. Effectiveness of the Decellularization Procedure

**Figure 2:**
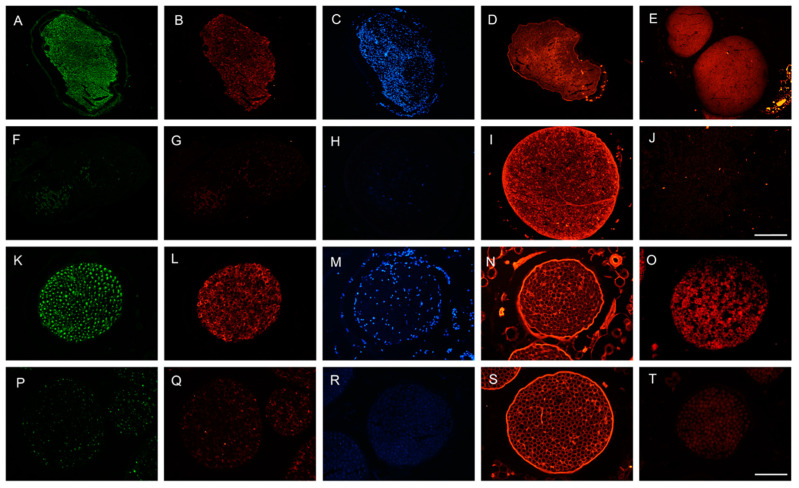
Representative micrographs showing immunofluorescence of myelinated axons (**A**,**F**,**K**,**G**), Schwann cells (**B**,**G**,**L**,**Q**), nuclei (**C**,**H**,**M**,**R**), extracellular matrix proteins (**D**,**I**,**N**,**S**) and myelin (**E**,**J**,**O**,**T**), in a control rat sciatic nerve (**A**,**B**,**C**,**D**,**E**), in a decellularized rat nerve (**F**,**G**,**H**,**I**,**J**), in a control sheep peroneal nerve (**K**,**L**,**M**,**N**,**O**) and in a decellularized sheep nerve (**P**,**Q**,**R**,**S**,**T**). Scale bar 150 μm.

The decellularization procedure developed removed all donor cells and DNA from the nerve segments, while the ECM as well as structural layers of the graft remained intact. In this way, the nerve grafts can provide guidance to the regenerating axons upon implantation. DNA content averaged 159.7 ± 18.7 ng/mg tissue in the native nerves of sheep, and 0.35 ± 0.02 ng/mg tissue in the decellularized nerves. Cross sections of decellularized nerve grafts were processed for immunohistochemistry before implantation. Immunolabeling against laminin showed that the structure of the ECM was well preserved. Faint staining against NF200, S100 and Fluoromyelin in the decellularized nerve grafts, of both rats and sheep, was compatible with lack of axons (NF200) and Schwann cells (S100), and good decellularization (absence of DAPI, general marker of nucleus), thus indicating that cell contents were completely removed (Figure 2).

### 3.2. Rat Study

**Figure 3:**
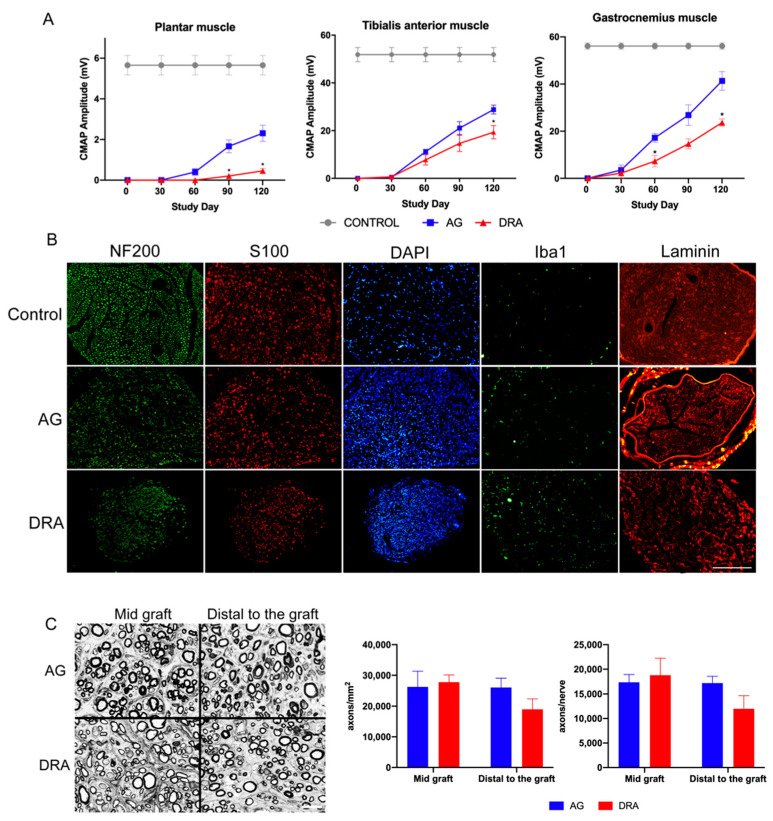
Results of the electrophysiological tests and histological evaluation after repair of a 15 mm long gap in the rat sciatic nerve. (**A**) Electrophysiological evaluation of nerve regeneration along 120 days follow-up after sciatic nerve section and repair with an autograft (AG, *n* = 5) or with a decellularized allograft (DRA, *n* = 5). Results are presented as mean ± SEM. Statistical analysis was performed using 2way ANOVA. Plots show the amplitude of CAMP of plantar (* *p* < 0.05 vs. AG), tibialis anterior (* *p* < 0.05 vs. AG) and gastrocnemius (* *p* < 0.05 vs. AG) muscles. (**B**) Representative micrographs showing immunofluorescence of myelinated axons labeled against Neurofilament 200, Schwann cells labeled against S100, nuclei labeled with DAPI, macrophages labeled with IBA1 and extracellular matrix labeled with laminin in cross sections of nerve graft in control, AG and DRA groups; scale bar 200 μm. (**C**) Representative transverse semithin sections of the mid graft and distal to the graft in AG and DRA groups, stained with toluidine blue. Scale bar 10 μm. Plots show the density and the number of myelinated axons in the sciatic nerve at mid graft and distal to the graft. Normal values in our laboratory for the sciatic nerve in rats average 8076 ± 215 myelinated axons, with a density of 11,336 ± 698 per mm^2^.

Denervation of the GM, TA and PL muscles was observed in both experimental groups at 30 dpi. At 60 dpi, all animals from the AG and DRA groups showed electrophysiological evidence of starting reinnervation in the GM and TA muscles. Three of the animals of the AG group had recorded CMAPs of small amplitude evoked by electrical stimulation, whereas none of the animals of the DRA group showed evidence of reinnervation in the PL muscle. At 90 dpi, the amplitude of the CMAPs increased for TA and GM muscles in both groups. All the rats of the AG group but only two thirds of the rats of the DRA group showed positive values for PL muscle. At the end of the follow-up, set up at 120 dpi, group AG had significantly higher mean CMAP amplitude values than the DRA group in TA (28.8 ± 1.8 mV vs. 19.3 ± 2.8 mV; *p* < 0.05), GM (41.3 ± 3.9 mV vs. 23.7 ± 1.5 mV; *p* < 0.05) and PL (2.3 ± 0.4 mV vs. 0.5 ± 0.1 mV; *p* < 0.05) muscles (Figure 3A).

The histological observations of transverse nerve sections showed the typical appearance of regenerated nerves, with numerous small size myelinated and unmyelinated axons inside the endoneurial compartments of the nerve graft and in the distal nerve. Immunohistochemical labeling showed numerous axons accompanied by Schwann cells along the grafts, but at lower density than in control nerves (Figure 3B). Quantitative analysis of the myelinated axons under light microscopy showed similar density in the middle of the graft in the AG group (26,320 ± 5013 axons/mm^2^) compared to the DRA group (27,858 ± 2257 axons/mm^2^), and similar total number of axons (17,395 ± 1525 axons/nerve) in the AG group and DRA group (18,773 ± 3464 axons/nerve). Distally to the graft, all animals from the AG and DRA groups had regenerated myelinated axons, but the density was higher in the AG group (26,105 ± 2991 axons/mm^2^) than in the DRA group (18,992± 3358 axons/mm^2^); similarly, the total number of axons distal to the graft was also higher in the AG group (17,224 ± 1329 axons/nerve) compared to the DRA group (11,983 ± 2664 axons/nerve) (Figure 3C), but without significant differences between the two groups.

### 3.3. Sheep Study

All sheep from both groups recovered well from the surgical procedure without any side effect and survived until the end of the study. No significant clinical signs, except for the right hindlimb dysfunction, were observed in the animals during the experimental follow-up. The sheep were able to stand and walk and have good mobility. Due to the peroneal nerve injury, two animals, one of each group, had a marked foot-drop posture that produced pressure ulcers on the dorsal skin of the paw. A splint was placed 30 days post-operation to prevent further foot lesions, and ulcers were treated with chlorhexidine and antibiotic cream and covered. Only one of the sheep, in group DC, did not recover and continued with the foot-drop posture until the end of the study.

#### 3.3.1. Functional Test Results

**Figure 4:**
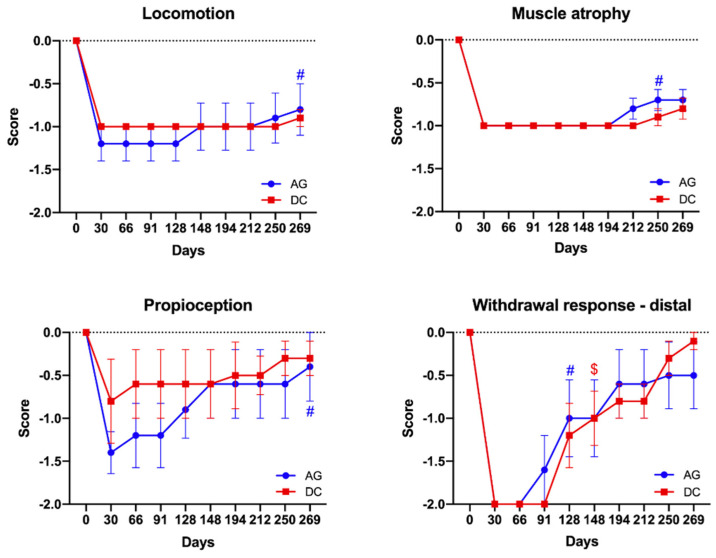
Plots of the functional evaluation along the 9 months follow-up in the two groups of sheep. Similar evolution was observed for all the tests applied, without significant differences between the groups AG and DC. Regarding the time evolution, AG group (*n* = 5) showed a significant recovery in all the tests applied (# *p* < 0.05) vs. baseline at 30 days post-operation, while animals of DC group (*n* = 5) only showed a significant recovery in the withdrawal response-distal ($ *p* < 0.05) vs. baseline at 30 days post-operation.

All the sheep showed close to normal locomotion activity after the surgery. In the resting orthostatic position, all the sheep, except one from the AG group, were able to correctly position the right hind hoof, maintaining the plantar support. When tested during fast walking, a foot-drop gait was evidenced in all sheep, as they failed to make plantar stepping in some steps (scored −1 or −2). A significant improvement (*p* < 0.05) in dorsiflexion was observed at the end of the follow-up in the AG group with respect to values at 30 days (Figure 4). The right TA muscle showed a reduction in size from one month after the surgery. There was a significative improvement appreciated in the last two months of the follow-up in the AG group. The reposition of the injured hindlimb when displaced to the dorsum, indicating proprioceptive inputs, was partially reduced after the surgery and the score significantly recovered to normal value at the end of the follow-up. The withdrawal reflex response was induced in 3 different sites: proximal, mid and distal in the dorsum of the paw. The response was suppressed after the surgery and slowly recovered with time. At the distal site, the test was more sensitive, without full recovery in all the sheep at 9 months post-operation (Figure 4). The AG group showed significant recovery from 4 months post-operation while the DC group had significant improvement from 5 months post-operation with respect to values at 30 days.

Overall, the functional tests showed the expected failure due to a peroneal nerve injury, and partial recovery of sensory-mediated functions, whereas motor recovery and muscle mass remained relatively steady until the last month of follow-up.

#### 3.3.2. Electrophysiological Test Results

**Figure 5:**
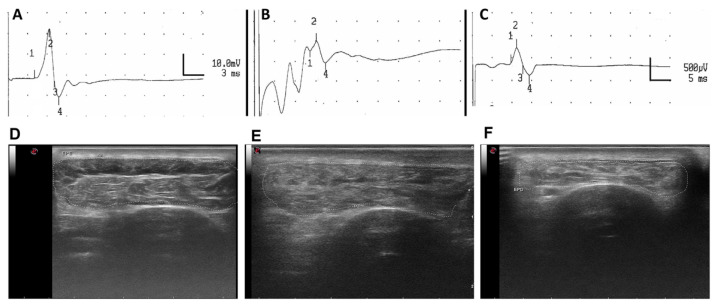
Representative EMG recordings of the CMAP recorded in the TA muscle evoked by stimulation of the sciatic nerve in the intact left hindlimb (**A**), and in the operated right hindlimb of a sheep at 6.5 (**B**) and 9 (**C**) months after operation. Labels: “1” at the onset, “2” at the peak, “4” at the end of the CMAP. Scale in A: amplitude 10 mV/square and time 3 ms per square; in **B** and **C**: amplitude 500 μV/square and time 5 ms per square. Representative echographic images of the TA muscle recorded in the intact left hindlimb (**D**) and in the operated right hindlimb of a sheep repaired with an autograft (**E**) or with a decellularized nerve allograft (**F**).

In the left, control hindlimb, the TA CMAP evoked by stimulation at the sciatic notch appeared at an onset latency of 4.2–4.4 msec and had a maximal amplitude of 20–22 mV, with small variations between sheep (Figure 5A). In the right, operated side, at 5 months none of the animals showed evidence of TA muscle reinnervation. Fibrillation potentials, signs of existing muscle denervation, were detected. At 6.5 months, CMAPs of late latency, small amplitude and disperse shape were recorded in 3 sheep of group AG and in 3 sheep of group DC. Fibrillation potentials were also recorded in TA muscles without CMAP response (Figure S3). At 9 months, CMAPs, recorded in 4 sheep of each group, were of slightly higher amplitude and still of long latency, compatible with early reinnervation of the TA muscle. No significant differences were found between groups AG and DC in any parameter of the nerve conduction tests, although CMAP amplitude was comparatively higher in group AG than in group DC (Table 1).

#### 3.3.3. Echographic Evaluation of TA Muscles

In denervated muscles, the density of the muscle imaging changed, and the size (measured both as area and perimeter) was decreased, evidencing muscle atrophy and increased connective tissue secondary to denervation (see Figure 5D–F). At 6.5 months post-surgery, the TA muscle area in the DC group was significantly lower than in the AG group (** *p* < 0.01). At the end of the follow-up, there was a tendency to recover muscle size, although it was still significantly lower in the DC group than in the AG group (* *p* < 0.05) (Table 1). The size of the TA muscles in the two sheep without electrophysiological responses, one from each experimental group, was the smallest (1.85 cm^2^ in area and 7.41 cm in perimeter in the AG group and 1.56 cm^2^ and 7.36 cm in the DC group), pointing to a relationship between muscle size and degree of reinnervation. 

#### 3.3.4. Histological Assessment

**Figure 6:**
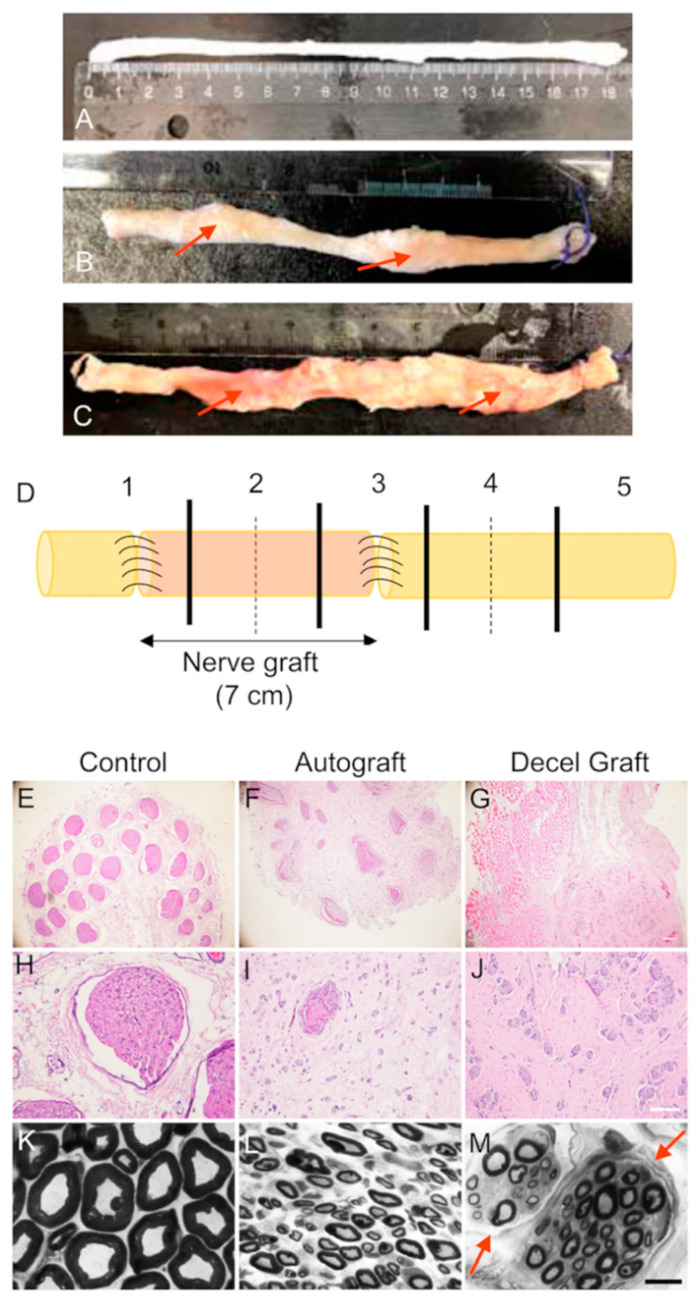
Macroscopic representative pictures of a control sheep peroneal nerve (**A**), a peroneal nerve autograft (**B**), and a decellularized nerve allograft (**C**), both with a neuroma visible (marked with red arrows) at proximal and distal sutures, harvested at the end of the study. (**D**) Schema of the division in sections of nerves harvested. Section 1 includes the proximal suture and Section 3 includes the distal suture of the graft. Section 2 and Section 4 were divided into two different segments (dotted line). Micrographs of cross sections of the proximal segment of the graft of sheep stained with hematoxylin and eosin. (**E**,**H**) control nerve, (**F**,**I**) autograft and (**G**,**J**) decellularized nerve graft; **E**, **F**, **G** scale bar 200 μm, and **H**, **I**, **J** scale bar 100 μm. Representative semithin transverse sections of the graft of sheep in AG and DC groups, stained with toluidine blue. (**K**) control nerve, (**L**) autograft and (**M**) decellularized nerve graft, scale bar 10 μm. Red arrows in M point to the newly formed regenerative small fascicles.

Most of the animals from AG and DC groups presented grafted nerves with a well-preserved structure and neuroma visible at proximal and distal ends corresponding with the suture lines (Figure 6A–C). The control peroneal nerve was composed of multiple small fascicles, usually more than 30, each containing numerous nerve fibers densely packed in the endoneurium. In the AG group, the structure of the fascicles was preserved in the graft, but regenerating axons were detected both inside and outside of the perineurium delimiting the fascicles. In contrast, no clear fascicles were observed in the decellularized grafts, where regenerating axons were seen grouped in small regenerative units, disperse within the graft structure (Figure 6E–M).

Semithin sections stained with toluidine blue showed, in the AG group, a preserved structure of the fascicles with many myelinated axons of different sizes. In addition, there were small new regenerative units that contained a small number of myelinated axons. In the DC group, the structure of the fascicles was not conserved, although new regenerative units were observed throughout the nerve with a large number of myelinated axons (Figure 6M; marked with red arrows).

**Figure 7:**
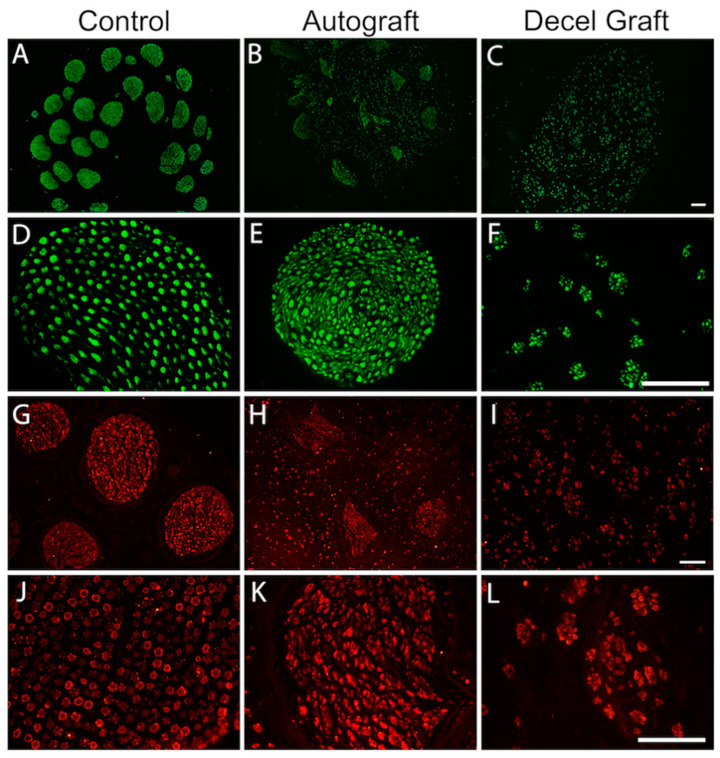
Representative micrographs showing immunofluorescence of myelinated axons labeled against Neurofilament 200 in cross sections of the proximal graft in AG and DC groups. (**A**,**D**) control nerve, (**B**,**E**) autograft and (**C**,**F**) decellularized nerve graft. **A**, **B**, **C** scale bar 200 μm, and **D**, **E**, **F** scale bar 100 μm. Representative micrographs showing immunofluorescence labeling of Schwann cells with an antibody against the S100 protein in cross sections of the proximal graft in AG and DC groups. (**G**,**J**) control nerve, (**H**,**K**) autograft and (**I**,**L**) decellularized nerve graft. **G**, **H**, **I** scale bar 100 μm, and **J**, **K**, **L** scale bar 100 μm.

Distal to the graft, the multifascicular structure of the peroneal nerve was maintained and most regenerating axons grew within the distal nerve fascicles. Immunohistochemical labeling at the middle part of the grafts (noted with dotted line in Figure 6D) from AG animals showed axons and Schwann cells (Figure 7) present within both fascicular and extrafascicular areas, whereas in the decellularized grafts of the DC group, axons and glial cells were dispersed in small regenerative clusters. Regarding the analysis of NF200 labeled axons, group AG had 1 animal with a very low number of regenerating axons while 2 animals had higher than control values. One animal did not show regenerated axons, likely attributable to suture dehiscence early after the operation. Group DC also had 1 animal without regenerating axons.

The estimated mean number of myelinated axons was 21,676 ± 3716 in the control peroneal nerve, whereas in the mid segment of the grafts (Figure 6 Section 2 dotted line) there were 26,213 ± 2798 axons in the four regenerated sheep of the AG group, and 18,724 ± 3410 in the four sheep of the DC group at 9 months post-surgery. The corresponding values at the nerve distal to the graft were 20,600 ± 6082 for the AG group and 7780 ± 1871 (*p* < 0.05) for the DC group (Figure 6 Section 4 dotted line). The higher number of regrowing axons in the graft is indicative of some excessive regenerative sprouting that was remodeled at the distal nerve.

#### 3.3.5. Histological Evaluation of Reinnervated Targets

**Figure 8:**
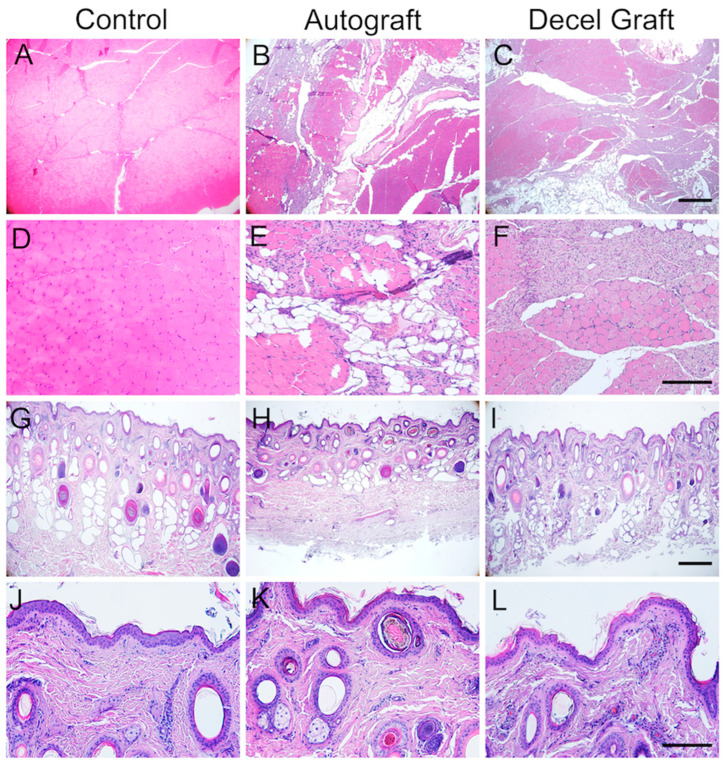
Representative micrographs of cross sections of the Tibialis Anterior muscle stained with hematoxylin and eosin from (**A**,**D**) the contralateral side (control), (**B**,**E**) an animal from AG group, (**C**,**F**) an animal from DC group. **A**, **B, C** scale bar 200 μm, and **D**, **E**, **F** scale bar 100 μm. Representative micrographs of perpendicular sections of the skin of the dorsum of the foot stained with hematoxylin and eosin from (**G**,**J**) the contralateral side (control), (**H**,**K**) an animal from AG group, (**I**,**L**) an animal from DC group. **G**, **H**, **I** scale bar 200 μm, and **J**, **K**, **L** scale bar 100 μm.

In the operated hindlimb, the TA muscle showed a heterogeneous structure in both groups, AG and DC (Figure 8A–F). Some areas appeared normal, although muscle fibers had a smaller diameter than normal, likely corresponding to reinnervated areas of the muscle. Other areas showed signs of atrophy and inflammatory infiltration. The skin samples, processed in sagittal sections, showed distinct organization in three layers: epidermis, dermis and subcutaneous tissue. The skin of the dorsum of the paw had a similar aspect to the skin from the contralateral side, without signs of cell infiltration or atrophy (Figure 8G–L).

## 4. Discussion

The gold standard repair technique for long gap peripheral nerve injuries is grafting an autologous nerve. However, sources for autologous nerve grafts are limited, so it is not possible to use this method to treat extended or multiple nerve lesions [29]. Acellular nerve allografts appear as a good alternative for autografts, considering that they come from a natural and abundant source, are not overtly immunogenic, and may keep a normal-like ECM structure which helps guiding regenerating axons [8,30]. Moreover, an allograft may offer several technical advantages over the autograft, such as off-the-shelf availability, easy handling, shorter surgical time, and no donor graft site morbidity [31]. Nevertheless, acellular allografts also have some limitations, mainly related to the lack of cell support for axonal regeneration, since it is important to eliminate cellularity in order to avoid immune rejection.

The results of our studies indicate that the used, novel procedure for tissue decellularization is highly efficient, eliminating all cells and DNA from the nerve segment, while preserving a normal-like structure of the perineurium and endoneurial tubules. Consequently, the decellularized graft did not induce an immune rejection response after in vivo implantation and proved to be a good substrate for axon growth and migration of host cells into the graft, allowing effective regeneration and functional recovery. The results of the two studies made in rats and in sheep showed promising results in terms of functional reinnervation and axonal regeneration, although the outcomes of the DC allograft were comparatively lower than those achieved with an ideal AG (i.e., the same nerve segment resected and re-sutured in place). The DC allograft allowed effective regeneration across a critical 15 mm long nerve gap in the rat sciatic nerve, and a 7 cm long gap in the sheep peroneal nerve, in both cases a length that cannot be effectively bridged with synthetic nerve conduits and represents a challenge for surgical nerve repair. The limiting gap size depends on the nerve and the animal species. Seminal experimental reports showed that regenerating axons are able to bridge synthetic conduits such as silicone, up to 4 mm in the mouse and 10 mm in the rat [15,32], and less than 30 mm in primates [33,34], but failed through longer gaps.

### 4.1. Nerve Regeneration in the Sheep Peroneal Nerve Model

Small animal models are commonly used to replicate challenging clinical scenarios [6,26,35,36]. In contrast to small-animal models, large-animal models allow investigation of longer and more clinically relevant nerve defects and harvesting long segments of donor nerves. This reflects the actual situation in most human nerve injuries in which damage to the nerve often extends over several centimeters [22]. Hence, standardized models of long nerve gap injury in large animals are necessary to evaluate tolerability and efficacy of new treatment strategies for severe nerve injuries [6]. In this sense, sheep are proposed as an optimal large animal model, since they have similarities in general body and neural structures to humans [37,38], and particularly peripheral nerve dimensions and structure similar to humans [20,39,40,41]. Moreover, their calm nature, compared to other large animals used as experimental models, such as pigs, enables easier postoperative management and clinical testing [18,42].

In the present study, the regenerative potential of a novel decellularized nerve allograft was evaluated and compared to autograft repair of a 70 mm long resection of the peroneal nerve in sheep. This nerve is the most commonly affected in injuries involving the lower limb in humans [41,43]. Moreover, it is adequate because it is similar in size and plurifasciular, as is the human peroneal nerve, and induces a limited motor deficit that still allows the animals to stand and walk [20,44]. Since the common peroneal nerve is a mixed nerve, both motor and sensory functional loss in the denervated targets is expected [45]. Therefore, paralysis of dorsiflexion muscles and lack of sensitivity of the dorsum of the foot was observed, although all the sheep showed close to normal locomotion after recovery from the surgery, and most of them were able to position the hind hoof in a normal resting position. Similar to motor function, proprioceptive sensibility of the denervated muscles was also affected, and complete recovery was achieved with time in all the animals. We hypothesize that this recovery could be accounted by the normal processing of information by the intact tibial and sural nerves, also innervating the hind limb, so that the contribution of the peroneal nerve is limited. The response observed as a withdrawal flexion of the hind limb at early times, when no axons of the injured peroneal nerve could have regenerated to the ankle level, can be attributed to collateral reinnervation by sprouts from nearby cutaneous nerve tributaries of tibial and sural nerves, a phenomenon that has been well studied in other smaller mammals [46,47] as well as in human subjects [48]. Therefore, results obtained in the functional sensory tests can be confounded by collateral reinnervation and are not completely useful to report nerve regeneration and cutaneous reinnervation. In contrast, electrophysiological assessment of the recovery of the CMAP of the TA muscle was the most sensitive test. Although the electrophysiological parameters rarely return to normal levels after nerve injury, they are frequently used to evaluate nerve regeneration and muscle reinnervation in humans and animal models [6,49,50,51]. Electrophysiological tests evidenced reinnervation of the TA muscle in a 3/5 sheep of both AG and DC groups at 6.5 months, and in 4/5 at 9 months. The time needed for axonal regeneration to distal targets in the sheep hindlimb is considerably long, taking into consideration that the distance between the proximal section of the peroneal nerve and the entrance of the nerve into the TA muscle is about 34 to 36 cm. Since we found early electrophysiological evidence of reinnervation of the TA muscle at 6.5 months, a regeneration rate between 1.7 and 2 mm/day can be estimated. The lower amplitude of the recorded CMAPs in the DG group than in the AG group, may suggest a slightly slower regeneration along the decellularized graft.

At the end of follow up, histological assessment of the graft and the distal regenerated nerve was performed. The extent of axonal regeneration in the grafts was evaluated by using neurofilament immunolabeling [22,52]. Schwann cells were observed within the decellularized nerve graft 9 months after the repair, indicating that the host cells migrated from proximal and distal stumps into the graft. Neurofilament labeling was present in most of the grafts, corroborating successful axonal regeneration in these animals and confirming the functional results. However, the incomplete functional recovery observed, as evidenced by the low amplitude of TA CMAPs recorded at 9 months (about 8% and 2% of control values in AG and DC groups, respectively), compared with the extensive amount of regeneration into the grafts suggests that many regenerating axons need more time to reach target organs and therefore, longer follow up periods are needed in this model to assess maximal functional outcomes, as reported also in humans [53].

### 4.2. Regenerative Potential of the Acellular Nerve Graft

The only available and FDA approved processed nerve allograft to repair human nerve defects of a length up to 5 cm is the marketed allograft Axogen Avance^®^. This product provides an off-the-shelf alternative to synthetic conduits while maintaining some proregenerative properties of autologous nerve grafts [54], providing overall 82% meaningful recovery in repairs of nerve gaps up to 70 mm [55]. No adverse events have been observed, despite some failures reported pointed to a careful consideration of acellular nerve allografts in long, large diameter injured nerves [56]. In this study, we have evaluated a novel decellularized nerve graft that allowed axonal regeneration across not only a 15 mm long gap in rats, but also a 70 mm gap in sheep, offering good perspectives for marketing applications. Electrophysiological and functional test results indicated a similar course, although with some delay, of muscle reinnervation than with the ideal autograft used.

However, the organization of the regenerating axons was different in the decellularized graft compared to the autograft. In the decellularized grafts, regenerated axons were found in an extrafascicular location, and only in the distal nerve sections the fascicles were consistently well defined, with very few extrafascicular axons visible, as described in previous studies [22,57]. This indicates that the matrix of the decellularized nerve is not so consistent as in a fresh nerve autograft, that has not been previously manipulated, even when the macroscopic observation indicates a well-preserved structure of the connective tissue, ECM molecules and fascicles in the decellularized grafts before implantation. Although further optimization of the decellularization protocol can be performed, there is a delicate equilibrium between preserving nerve architecture and completely decellularizing the graft to avoid immune rejection.

Finally, the number of regenerated axons distal to the graft was lower in the DC group compared to the AG group, indicative of a slower regeneration along the decellularized graft. Therefore, although the decellularized graft is permissive for regeneration, the lack of a cellular component causes slower regeneration into the graft compared to the ideal cellular autografts. This is expected, mainly due to the important role of Schwann cells in axonal regeneration [8]. Interestingly, this slower regeneration into the graft implies a delayed onset but does not have to translate in a significantly poorer functional recovery, according to previous observations [9]. In fact, electrophysiological recovery was quite limited in the two groups of rats followed for 4 months (see Figure 3A), and in the two groups of sheep evaluated up to 9 months after injury (Table 1), comparing to control values obtained in the contralateral hind limb. These results point out the long gap bridged by the graft and the long distance that axons had to grow to reinnervate target organs as determinants for the functional outcome. In this sense, the optimization of acellular grafts and recellularization methods to improve their regenerative capability offers a window of opportunity to improve recovery in this type of injuries, when the gold standard technique has already limited potential.

## Figures and Tables

**Table 1:** Values of the nerve conduction tests in the peroneal nerve and of the echography tests in the peroneal innervated muscles, in the control left limb and in the operated right limb (*n* = 5 each) at 6.5 and 9 months postoperation. * *p* < 0.05 vs. AG; ** *p* < 0.01 vs. AG. Results were analyzed by unpaired *t* test using GraphPad Prism 8 software.

Nerve Conduction.	Group	Latency (msec)	CMAP Amplitude (mV)	n + Response
Control (L)	AG	4.38 ± 0.16	21.26 ± 1.54	5/5
Control (L)	DC	4.24 ± 0.09	22.84 ± 1.02	5/5
6.5 mo	AG	11.05 ± 1.52	0.97 ± 0.48	3/5
6.5 mo	DC	16.87 ± 4.54	0.21 ± 0.11	3/5
9 mo	AG	11.58 ± 1.06	1.87 ± 0.72	4/5
9 mo	DC	13.16 ± 1.50	0.46 ± 0.16	4/5
**Echography**		**TA area (cm^2^)**	**TA perimeter (cm)**	
Control (L)	AG	5.30 ± 0.29	10.30 ± 0.24	
Control (L)	DC	5.31 ± 0.48	10.30 ± 0.44	
6.5 mo	AG	1.90 ± 0.07	7.34 ± 0.23	
6.5 mo	DC	1.48 ± 0.03 **	6.87 ± 0.08	
9 mo	AG	2.44 ± 0.21	7.95 ± 0.17	
9 mo	DC	1.85 ± 0.09 *	7.45 ± 0.11	

## Data Availability

The data presented in this study are available on reasonable request from the corresponding author.

## Supplementary Materials

The following supporting information can be downloaded at https://www.mdpi.com/article/10.3390/cells11244074/s1, Figure S1: Flexor withdrawal reflex test; Figure S2: Performance of the echography of the Tibialis Anterior muscle; Figure S3: Fibrillation potentials.
