# Supplementary material for: Repair of Long Nerve Defects with a New Decellularized Nerve Graft in Rats and in Sheep
Source: Cells. 2022 Dec 16;11(24):4074. doi: 10.3390/cells11244074 (PMC9777287; doi:10.3390/cells11244074)

Supplementary Figure 1: Flexor withdrawal reflex test. Photograph of the maneuver used to pinch the dorsum of the foot with a hemostat for assessing pain sensitivity and the induced withdrawal response. The pinch was applied at a proximal (pointed by arrow), a mid (arrow) and a distal point (hemostat) between ankle and hoof.

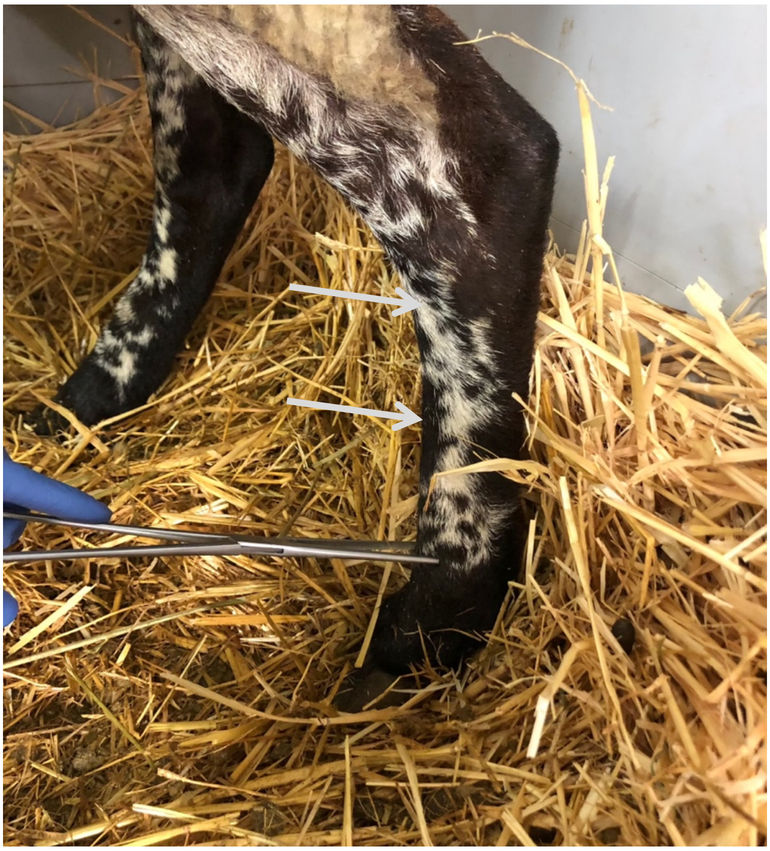

Supplement: Supplementary file 1 [file cells-11-04074-s001.zip › Supplementary Figure S1 .pdf]
