# Supplementary material for: Repair of Long Nerve Defects with a New Decellularized Nerve Graft in Rats and in Sheep
Source: Cells. 2022 Dec 16;11(24):4074. doi: 10.3390/cells11244074 (PMC9777287; doi:10.3390/cells11244074)

Supplementary Figure 2: Performance of the echography of the Tibialis Anterior muscle. Photograph of the hindlimb with the handheld ultrasound probe placed at a midpoint between the tibial crest (marked with a red *) and the tuber calcanei to obtain a cross-sectional image of the TA muscle. Before image acquisition, the skin was shaved, and cleaned with mild soap and water. Ultrasound gel was placed on the probe contact surface.

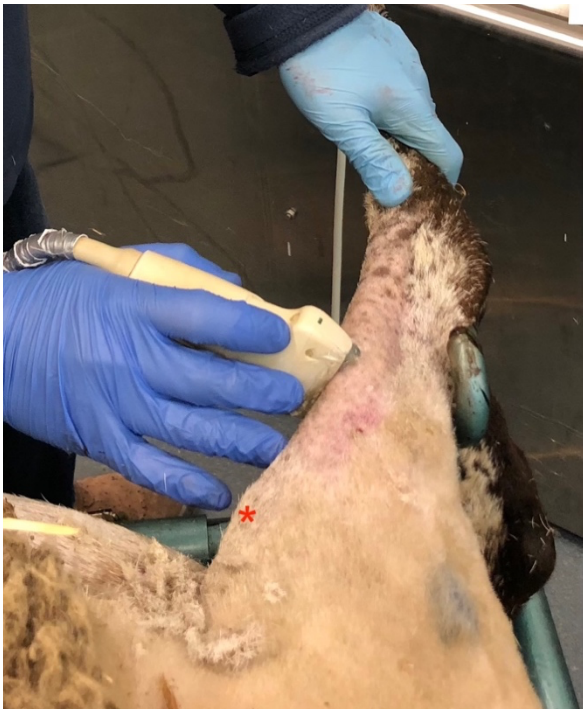

Supplement: Supplementary file 1 [file cells-11-04074-s001.zip › Supplementary Figure S2 .pdf]
