# Supplementary material for: Repair of Long Nerve Defects with a New Decellularized Nerve Graft in Rats and in Sheep
Source: Cells. 2022 Dec 16;11(24):4074. doi: 10.3390/cells11244074 (PMC9777287; doi:10.3390/cells11244074)

Supplementary Figure 3: Fibrillation potentials. Recordings of fibrillation potentials in the denervated TA muscle of the operated right hindlimb of sheep at 6.5 months after operation, repaired with an autograft (left trace) or with a decellularized nerve allograft (right trace).

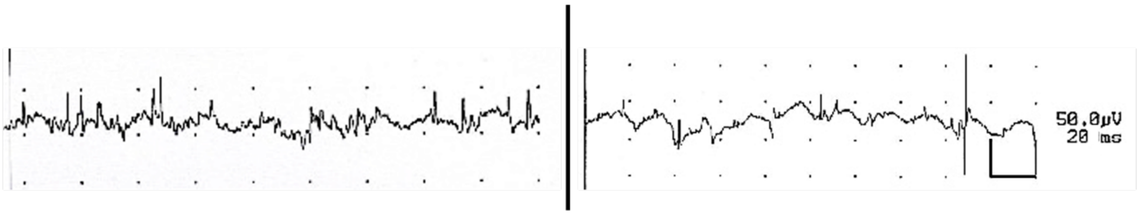

Supplement: Supplementary file 1 [file cells-11-04074-s001.zip › Supplementary Figure S3.pdf]
